# A case report of hypocomplementemic urticarial vasculitis presenting with membranoproliferative glomerulonephritis

**DOI:** 10.1186/s12882-020-02001-6

**Published:** 2020-08-18

**Authors:** Kalliopi Vallianou, Chrysanthi Skalioti, George Liapis, John N. Boletis, Smaragdi Marinaki

**Affiliations:** 1 Department of Nephrology and Renal Transplantation Unit, Faculty of Medicine, Laiko Hospital, National and Kapodistrian University of Athens, Athens, Greece; 2 Pathology Department, Laiko Hospital, Athens, Greece

**Keywords:** Complement, Case report, Hypocomplementemic urticarial vasculitis syndrome, Renal disease, Vasculitis

## Abstract

**Background:**

Hypocomplementemic urticarial vasculitis syndrome is an infrequent condition characterized by ocular, renal, gastrointestinal and pulmonary involvement with low serum complement levels and autoantibodies. Renal manifestations vary from microscopic hematuria to nephrotic syndrome and acute kidney injury. Accordingly differing histologic patterns have been reported.

**Case presentation:**

We present the case of a 65 years old woman with a history of chronic uveitis who presented with arthralgias, urticarial rush, nephrotic syndrome, glomerular hematuria and low serum complement. Kidney biopsy revealed an immune-complex membranoproliferative glomerulonephritis. The patient received induction therapy with steroids, cyclophosphamide and hydroxychloroquine followed by rapid clinical improvement and remission of proteinuria. Maintenance treatment consisted of rituximab pulses.

**Conclusions:**

The majority of hypocomplementemic urticarial vasculitis syndrome cases is idiopathic, although an association to drugs, infections or other autoimmune disorders has been recorded. Given the rarity and heterogeneity of the disease, no standard treatment is established.

## Background

Hypocomplementemic urticarial vasculitis syndrome (HUVS) is a rare condition characterised by consistent urticarial lesions with histologic findings of leukocytoclastic vasculitis, as well as low serum complement levels and usual ocular, renal, gastrointestinal and pulmonary involvement. It represents the most severe of three distinct syndromes of the urticarial vasculitis (UV) spectrum. Normocomplementemic urticarial vasculitis (NUV) is a benign cutaneous vasculitis and hypocomplementemic urticarial vasculitis (HUV) is a low complement vasculitis also limited to the skin. HUVS, that is characterized by systemic involvement and usually circulating anti-C1q antibodies, was first described by McDuffie et al. in 1973 and the diagnostic criteria were defined in 1982 by Schwarz et al. Urticarial vasculitis is a rare disorder, with an incidence of 0,5/100.000persons, with only 1–2% of them, usually middle-aged women, developping the complete syndrome [1, 2]. It comprises a type III hypersensitivity reaction mediated by immune complex deposits on capillaries and postcapillary venules. Although most cases are idiopathic, the syndrome may be secondary to drugs, infections or other autoimmune disorders [3]. Given the limited number of cases and the heterogeneity of the symptoms, no standard treatment is established. The therapeutic options include corticosteroids, either as monotherapy or in combination with immunomodulatory or immunosuppressive agents [4].

## Case presentation

**Fig. 1:**
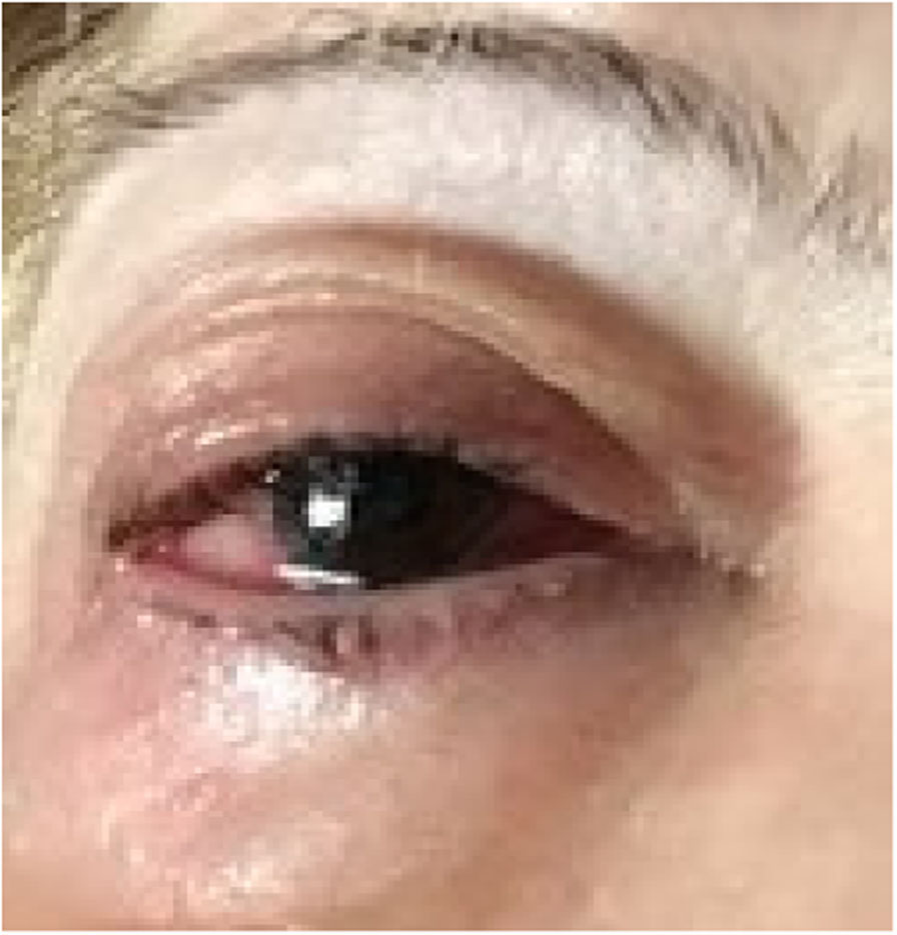
Ocular inflammation in our patient

The patient, a 65-year-old Caucasian woman, had a history of refractory chronic urticaria. She was treated by an allergiologist with omalizumab, an anti-IgE monoclonal antibody, for more than 3 years, but showed poor response. She also suffered from recurrent chronic uveitis for the last 2 years (Fig. 1) and complained about arthralgias. Six months before referral to our clinic, she developed isolated microscopic hematuria, followed 2 months later by subnephrotic proteinuria. Gradually, proteinuria increased to nephrotic level and she presented to us with nephrotic syndrome, with edema and hypalbuminemia.

Her medical history consisted of hypertension and type II diabetes mellitus, both diagnosed 10 years ago. Hypertension was well-managed with 5 mg of amlopidine, 160 mg of valsartan and 2,5 mg of nebivolol and diabetes with 1.700 mg of metformin daily. She also had Hashimoto’s disease, on levothyroxine for the last 20 years and was an active smoker (45 pack/years).

Οn examination remarkable were the urticarial rashes on her face, neck, torso and extremities that she described as rather pruritic than painful. They were refractory to antihistamines and resolved after a few days, leaving, hyperpigmentation of the skin. Physical examination also revealed tender tibial edema and arthritis of the small joints of the hands.

Serum creatinine was normal (0.9 mg/dl). Microhematuria of glomerular origin and nephrotic protinuria (6,6 g/d), with hypalbuminemia (3 g/dl) were also present. Inflammatory markers were high and complement components low, namely C1q, C4 and particularly C3 (Table 1).

**Table 1:** Laboratory results on diagnosis

		Normal values
Hemoglobin (g/dl)	10.8	12–16
WBC (/μl)	7.120	4.500–11.000
ptl (× 10^3^/μl)	505	140–440
ESR (mm)	70	< 10
CRP (mg/l)	38	
creatinine (mg/dl)	0,9	0,6-1,0
eGFR (ml/min/1,73m^2^)	67	> 60
albumin (g/dl)	3	3,5-5,0
TSH (μU/ml)	2,6	0,3-4,2
HbA1c (%)	5	< 5,7
**Immunological results**		
ANA	Neg.	Neg.
anti-dsDNA (IU/ml)	1,3	< 7
p-ANCA (AU/ml)	1,8	< 6
c-ANCA (AU/ml)	0,2	< 6
C1q (mg/dl)	35	34–65
C3 (mg/dl)	60	90–180
C4 (mg/dl)	10,4	10–40
cryoglobulins	Neg.	Neg.
**Viral serology**		
HbSAg	0,22	< 0,8
antiHCV	0,1	< 0,8
antiHIV	0,1	< 1,0
**Urinalysis**		
WBC	0–2	0–2
RBC	30–35	0–2
erythrocyte casts	Neg.	Neg.
protein	500	
glucose	Neg.	
24 h protein excretion (g/d)	6,6	< 0,15

Eye examination was positive for bilateral chronic anterior uveitis. Pulmonary function tests led to the diagnosis of chronic obstructive pulmonary disease (COPD), with FEV1 (forced expiratory volume in 1 s) 51%, FEV1/FVC (forced vital capacity) 60,5% without reversibility. It should be pointed out that the patient underwent two skin biopsies during the previous months, with findings compatible with common urticaria.

**Fig. 2:**
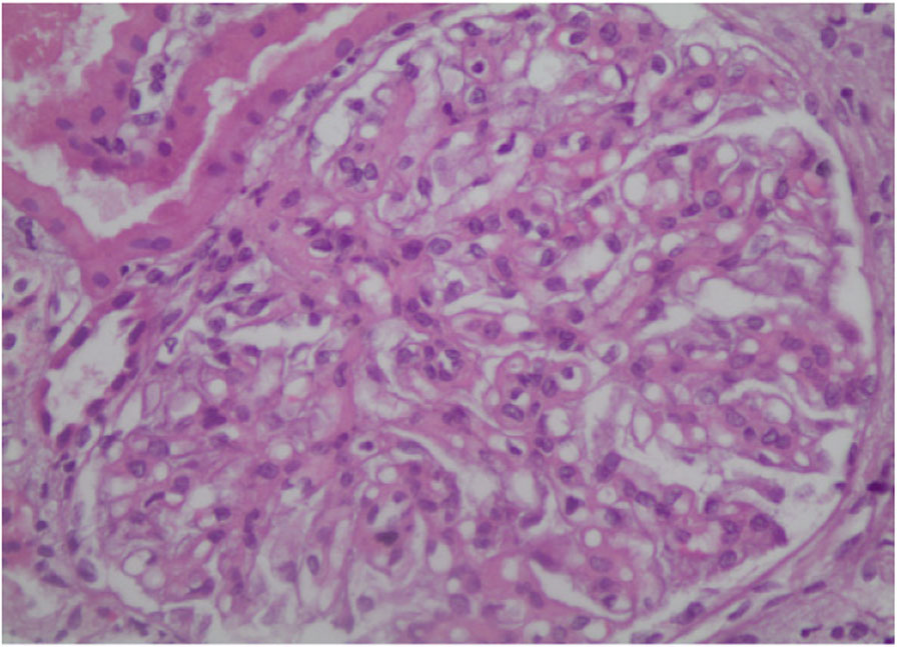
On light microscopy: Segmental mesangial proliferation with mild to moderate glomerular basement membrane thickening (H&E X400)

**Fig. 3:**
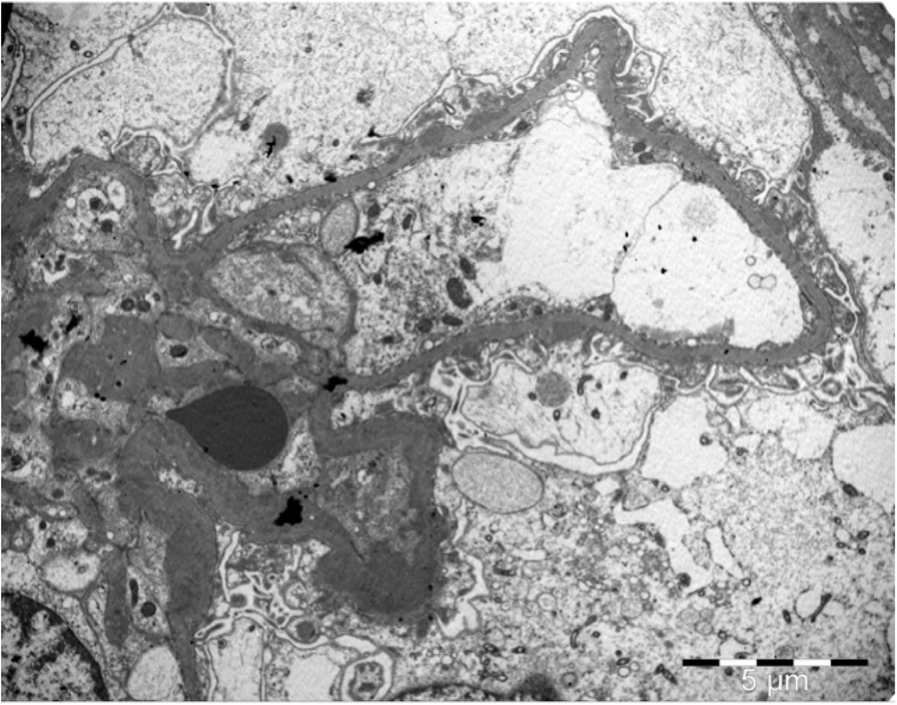
Subepithelial electron dense deposits along glomerular basement membranes, in association with mesangial deposits (Uranyl acetate X400)

Subsequently, an ultrasound-assisted percutaneous kidney biopsy revealed an immune-complex membranoproliferative glomerulonephritis. Light microscopy displayed 9 glomeruli, none of which was sclerosed. Mesangial matrix was segmentally increased, in association with mesangial cell proliferation. Glomerular basement membranes (GBM) exhibited focal thickening. Mild segmental thickening of the glomerular basement membrane (GBM) was seen (Fig. 2). In immunofluorescence examination, in scale intensity 0–4+, IgG and IgM immunoglobulins showed 3+ deposition with a granular pattern in the mesangium and focally along GBMs (subendothelial and subepithelial space), while IgA showed trace. C3 complement component showed 3+ with a similar pattern with IgG and IgM, while trace for C1q complement component was seen. Light chains, κ and λ showed an intensity of 3+ each, along GBM. Electron microscopy was also performed and demonstrated numerous electron-dense deposits mainly in the mesangium and less frequent in the subendothelial space, as well as subepithelial deposits. Focal podocyte foot process effacement was also observed (Fig. 3).

In this context, the diagnosis of hypocomplementemic urticarial vasculitis syndrome was established. Indeed, the patient fulfilled both major criteria, namely urticaria for more than 6 months and hypocomplementemia, and three of the six minor criteria which were arthritis, renal involvement and ocular inflammation.

**Fig. 4:**
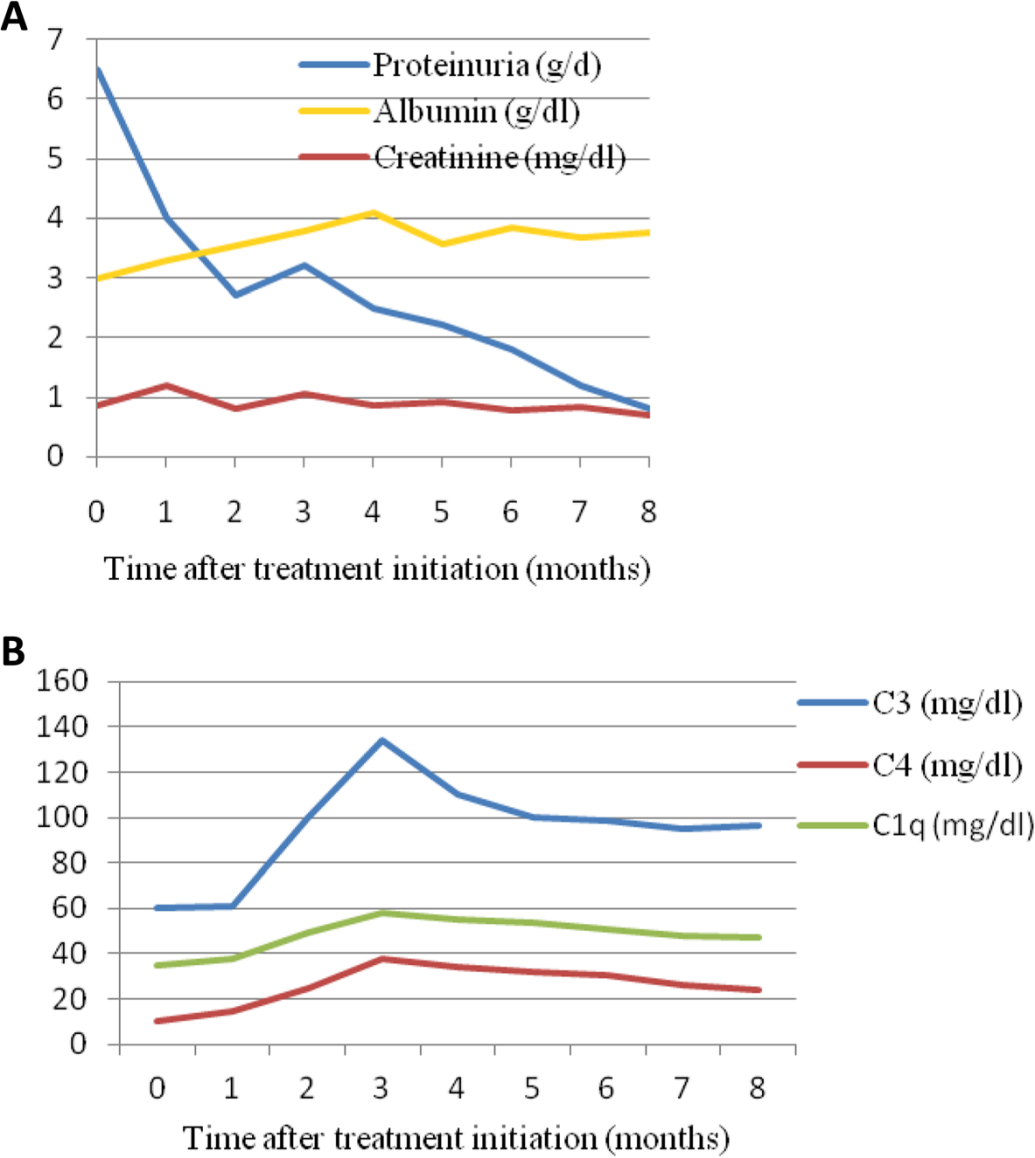
Patient’s response to treatment in relation with time: **a**. Serum creatinine, serum and urine albumin and **b**. C3 (normal 80–190 mg/dl), C4 (normal 10–40 mg/dl) and C1q (normal 34–65 mg/dl) complements

Induction treatment with steroids, cyclophosphamide and hydroxycholoquine was subsequently initiated. The patient received three daily intravenous pulses of 500 mg methylprednisolone, followed by 30 mg oral methylprednisolone daily (0,5 mg/kg prednisolone) with gradual tapering to 4 mg over a period of 5 months, six monthly pulses of cyclophosphamide (500 mg/m^2^) and 400 mg of hydroxychloroquine daily. Omalizumab and antihistamines were discontinued. The patient’s response to therapy was quick and sustained. The urticarial rash and arthralgias disappeared spectacularly a few weeks after treatment initiation. Uveitis relapsed two times since, but remains under control with local treatment. Renal and immunological indices normalized in the course of time (Fig. 4). Maintenance therapy consisted of rituximab 500 mg on Day 0 and 15, and every 6 months thereafter until month 18.

Eight months after diagnosis, the patient is normotensive, without skin presentations, stable renal function 24 h proteinuria of 800 mg.

## Discussion and conclusion

Urticarial vasculitis is a rare form of leukocytoclastic vasculitis. It can be normocomplementemic or hypocomplementemic and, when not limited to the skin, but also presenting with systemic manifestations, it is referred as hypocomplementemic urticarial vasculitis syndrome (HUVS) [5]. It is thought to be a continuum among these conditions. Patients often complain about rash or arthralgias for months before diagnosis; however it is remarkable how it took our patient 3 years to gradually develop the syndrome.

Urticarial rash, persistent or recurrent for more than 6 months, is present in all patients. Angioedema is common as well, present in about the half. Constitutional symptoms, such as fever, fatigue and malaise are rather rare, while arthralgias and arthritis are frequent. Common manifestations further include uveitis and abdominal pain, as well as renal and pulmonary involvement, each of them affecting 20–50% of patients [1, 6, 7]. Involvement of the heart, mainly pericarditis and valvular disease, is unusual, though possibly life threatening [8, 9]. Noteworthy are the pulmonary implications of the disease. Chronic obstructive pulmonary disease is common, usually emphysema, develops early at 4th or 5th decade and seems to result from the interaction between smoking and immune-complex mediated inflammation, although non-smokers can be affected as well [10, 11]. Overall HUVS is a condition with benign course. Morbidity and mortality is mostly associated with COPD severe renal or cardiac involvement*.* Most studies mention sporadic deaths, mainly associated with severe COPD, complications of end stage renal disease or infections, probably complications of immunosuppressive treatment [1, 10]. Exception is the report of Wisnieski et al. that 6 out of 18 patients died of respiratory insufficiency [11], There are reports of patients who underwent lung transplantation as well [1, 11]. Diagnosis is based on the Schwarz criteria (Table 2) [5]. Our patient met both major and three of the six minor criteria (arthritis, glomerulonephritis, uveitis).

**Table 2:** Schwarz criteria for the diagnosis of hypocomplementemic urticarial vasculitis

Major criteria	Chronic urticaria for more than 6 months
	Hypocomplementemia
Minor criteria	Leukocytoclastic vasculitis on skin biopsy
	Arthralgias or arthritis
	Ocular inflammation
	Glomerulonephritis
Modified by	Abdominal pain
Schwarz et al. [5]	Low C1q with positive anti-C1q antibodies

One interesting feature of HUVS is the heterogeneity regarding renal involvement. In a recent review of the literature, Boyer et al. identified 82 cases of HUVS with renal involvement, 88% of them being glomerulonephritis [12]. Isolated microscopic hematuria and subnephrotic-range proteinuria are common manifestations, whereas nephrotic syndrome and acute kidney injury are less common. Various histologic patterns have been described as well. The commonest histologic patterns are membranoproliferative and mesangioproliferative glomerulonephritis, followed by membranous nephropathy. Cases of minimal change disease, tubulointerstitial nephritis and rapidly progressive glomerulonephritis have also been described. No plausible explanation has been suggested to justify this heterogeneity and the factors affecting it [3, 12, 13]. Urticarial vasculitis is mediated by antigen-antibody complexes that are deposited on vascular lumina, resulting in complement activation through the classical pathway. The complement cascade, when triggered, releases chemotactic factors, which in turn attract inflammatory cells, resulting in the damage of the endothelium and surrounding tissues. The stimulus for these events seems to be activation of the C1q component. This explains why low C1q levels are found in the majority of patients (90–100%) with urticarial vasculitis. A suggested mechanism for the activation is binding of anti-C1q antibodies to the collagen-like region of the C1q molecule. Anti-C1q circulating antibodies are found in more than half of the patients and are, along with low C1q levels, considered to be the marker of the disease [5, 6, 11, 12]. Apart from these, other factors that could possibly activate C1q are drugs, like the anti-TNF factor etanercept [14], viruses, like Epstein-Barr and Hepatits B,C [15] and other, non-identified autoantibodies [15, 16]. Unfortunately, we could not test for anti-C1q antibodies, as the assay is not available not only in out hospital, but in any public laboratory in Greece. C1q and C4 levels were at the lowest end of the normal range. Therefore, we considered them low, particularly when combined with low C3 and her clinical presentation.

The uncertainty about the disease’s pathogenesis does not facilitate the differential diagnosis from other conditions, particularly systemic lupus erythematosus and cryoglobulinemia. It is not difficult to distinguish urticarial vasculitis from common urticaria, because the skin lesions last more than 24 h, usually 2–3 days, are sometimes painful or “burning” and may leave a residual bruising or hyperpigmentation of the skin [5, 17]. Skin biopsy typically reveals a small-vessel leukocytoclastic vasculitis that involves post-capillary venules. However, it seems that non-specific findings, such as lymphocyte and eosinophil infiltrates are relatively common, when an ‘older’ lesion is biopsied. Often, multiple biopsies of are needed to establish diagnosis of leukocytoclastic vasculitis, preferably of a wheal less than 12 h after eruption [18–20]. This is, in our opinion, the reason why our patient’s skin biopsies were negative for vasculitis and, considering that she already fulfilled Schwarz criteria, we chose not to put her through a third biopsy.

Differential diagnosis from lupus erythematosus is still a controversial issue. By many HUVS is thought to be a precursor or atypical presentation of lupus, taking into consideration the fact that there are patients presenting with urticarial vasculitis in whom the diagnosis of lupus is following after months or years. On the other hand, there are clinical and immunological differences showing that we are dealing with two distinct conditions. Lupus was on our differential diagnosis, considering the fact that our center has great experience with lupus patients and atypical manifestations of the disease. However, on immunofluoroescence, the classically described for the diagnosis of lupus nephritis “full-house” pattern was practically absent. Furthermore, in means of systemic manifestations, our patient did not fulfill the American Society of Reumatology (ASR). The typical butterfly rash does not occur in HUVS patients, while wheals and angioedema are rare in lupus. Ocular involvement in not so frequent in lupus, while pulmonary disease is particularly rare, in comparison to HUVS patients, in whom chronic obstructive pulmonary disease is a common finding. Renal involvement tends to be more common in lupus, kidney biopsy sometimes fails to distinguish between the two entities. Likewise, about half of vasculitis patients are positive for antinuclear antibodies, even though anti-dsDNA and ENA antibodies rarely are positive. Anti-C1q antibodies, are also found positive in 30% of lupus patients, as well as in other autoimmune diseases like Sjogren syndrome [3, 6, 16].

Given the rarity of the disease and the wide range of manifestations, treatment guidelines are not available as well. The various therapeutic options and their efficacy are summarized in a recent systematic review of 789 patients with urticarial vasculitis by Kohlkir et al. [4]. Corticosteroids are most widely used and lead to remission of skin lesions in more than 80%. Immunosuppressive and immunomodulatory factors have been used to enable steroid tapering. In cases limited to the skin hydroxychloroquine, dapsone, colchicine have been used with satisfactory response, while in those with systematic disease, agents like methotrexate, azathioprine, mycophenolate mophetil, cyclophosphamide, tocilizumab, anti-IL1 factors, cyclosporin and, lately, rituximab are preferred*.* However, in patients with more severe, disease the combination of steroids and cyclophosphamide remain the most efficacious option. Notably, antihistamines fail to relieve urticarial lesions, because urticarial vasculitis is a not a type I, but a type III hypersensitivity reaction [4–6]. Omalizumab can control normocomplementemic UV, but has contradictory results on hypocomplementemic UV [4, 21, 22].

In our case, the patient received induction with steroids and cyclophosphamide to ensure quick and stable remission of the disease and prevent complications associated with nephrotic syndrome. Taking into account the patient’s comorbidities, the drug’s toxicity and the fact that she had severe, but not life-threatening disease, we used a modest dose of cyclophosphamide (500 mg/m2). As for maintenance, we considered giving mycophenolate mophetil and rituximab, but decided on the latter. Both have been used on a small number of patients with comparable satisfactory response rates [4–6]. However, patients treated with rituximab seemed to sustain remission longer and suffered from fewer relapses [4, 6].

In conclusion, we present a case of hypocomplementemic urticarial vasculitis with nephrotic syndrome due to membranoproliferative glomerulonephritis, from the perspective of nephrologists who encountered this entity for the first and perhaps the last time. We find interesting that a patient initially diagnosed with chronic urticaria, gradually developed signs and symptoms like arthralgias, microscopic hematuria and uveitis, which could be easily ignored or missed. This underlines how important it is to closely monitor the patient and re-evaluate when new symptoms arise or treatment fails, like treatment with omalizumab in our case. We should always have in mind less usual entities like HUVS and, then, interdisciplinary cooperation is essential. Not only are HUVS cases few, there is also such a great heterogeneity among affected systems and symptoms, that we are given the impression that every case has a unique set of manifestations. This makes diagnosis more challenging, as we experienced with skin biopsies and explains the heterogeneity among treatments. Recent reviews have shed some light on treatment options and their efficacy. We think that applying this knowledge to the special features of every patient will help optimizing treatment.

## Data Availability

The datasets used and/or analyzed during the current study are available from the corresponding author on reasonable request.

## Abbreviations

HUV: Hypocomplementemic urticarial vasculitis
COPD: Chronic Obstructive Pulmonary Disease
GBM: Glomerular Basement Membrane
GFR: Glomerular Filtration Rate
FEV1: Forced expiratory volume in 1 s
FVC: Forced vital capacity
NUV: Normocomplementemic urticarial vasculitis
CKD-EPI: The Chronic Kidney Disease Epidemiology Collaboration
UV: Urticarial vasculitis
